# Pharmacokinetic Drug–Drug Interaction Potential of Oral Anticancer Drugs

**DOI:** 10.1002/cpt.70253

**Published:** 2026-03-07

**Authors:** Fatimah Alhurayri, Islam R. Younis, Shadia I. Jalal, Theodore F. Logan, Rohan Maniar, Steven M. Bray, Sara K. Quinney, Zeruesenay Desta, Todd C. Skaar, James E. Tisdale, Tyler Shugg

**Affiliations:** ^1^ Department of Pharmacy Practice Purdue University College of Pharmacy West Lafayette Indiana USA; ^2^ College of Pharmacy, Imam Abdulrahman Bin Faisal University Dammam Saudi Arabia; ^3^ Department of Quantitative Pharmacology & Pharmacometrics Merck & Co. Rahway New Jersey USA; ^4^ Division of Hematology/Oncology, Department of Medicine Indiana University School of Medicine Indianapolis Indiana USA; ^5^ Fountain Life, Inc. Indianapolis Indiana USA; ^6^ Division of Clinical Pharmacology, Department of Medicine Indiana University School of Medicine Indianapolis Indiana USA

## Abstract

Drug–drug interaction (DDI) management is critical for safe and effective use of oral anticancer drugs (OADs). Our study objectives were to (i) compile clinically relevant pharmacokinetic (PK) DDI mechanisms for OADs and (ii) assess the prevalence of PK potential DDIs (PDDIs) in patients with advanced solid cancers. OADs approved through January 2023 were assigned DDI mechanisms based on studies obtained from drug labels and primary literature showing ≥ 2‐fold exposure change or significant adverse health outcomes during co‐administration with interacting drugs. Electronic health records of 3,697 solid cancer patients were reviewed retrospectively to detect PDDIs, defined as overlapping prescriptions of OADs with relevant interacting drugs. FDA labels were reviewed for 99 OADs, and 239 studies were extracted from the primary literature, yielding a total of 748 drug–drug pairs for analysis. Eighty‐five OADs (85.9%) had ≥ 1 DDI mechanism. The most common DDI mechanisms were victims with metabolic inducers (71.7%), CYP3A substrates (55.6%), CYP3A perpetrators (29.3%), and victims with acid reducers (17.2%). Our primary literature search detected clinically relevant DDI mechanisms without actionable recommendations in the drug labels for 14 drugs. Among patients prescribed ≥ 1 OADs (46.9%), 17.4% had ≥ 1 PDDI, most commonly involving OADs acting as CYP3A (58.9%) or CYP2D6 (32.5%) perpetrators. Most OADs (~86%) had ≥ 1 DDI mechanism, and ~17% of solid tumor board patients had a clinically relevant PDDI.

**Study Highlights**

**WHAT IS THE CURRENT KNOWLEDGE ON THE TOPIC?**

Oral anticancer drugs have improved the efficacy and convenience of hematology/oncology patient care; however, drug–drug interactions (DDIs) are known to impact the efficacy and safety of many oral anticancer drugs. Due to the dynamic cancer therapeutic market and the multitude of concomitant medications taken by cancer patients, it is a struggle for even the most clinical pharmacology‐savvy clinicians to stay abreast of clinically significant DDIs impacting cancer care.

**WHAT QUESTION DID THIS STUDY ADDRESS?**

This investigation (i) comprehensively assessed the pharmacokinetic DDI mechanisms for FDA‐approved oral anticancer drugs through January 2023 using drug labels and the primary literature and (ii) characterized the prevalence of potential DDIs in a cohort of patients with advanced solid cancer.

**WHAT DOES THIS STUDY ADD TO OUR KNOWLEDGE?**

This study demonstrates that ~86% of oral anticancer drugs have at least one clinically relevant DDI mechanism. Additionally, ~17% of advanced solid cancer patients had potential DDIs likely to impact at least one drug therapy.

**HOW MIGHT THIS CHANGE CLINICAL PHARMACOLOGY OR TRANSLATIONAL SCIENCE?**

By comprehensively characterizing DDI mechanisms and providing therapeutic substitution recommendations to avoid DDIs, this manuscript may serve as a reference to oncology clinicians to reduce the frequency and clinical burden of DDIs in their patients.

Drug–drug interactions (DDIs) are of particular clinical importance in cancer patients, given the narrow therapeutic indices and the cellular toxicities of many anticancer medications.^1^, ^2^ Concurrent use of multiple medications (i.e., polypharmacy) is frequent in cancer patients, including medications to treat cancer and to manage cancer therapy‐related adverse events, cancer‐related symptoms, and comorbid conditions; this high frequency of polypharmacy confers an increased risk of DDIs, which can impact both the efficacy and toxicity of cancer treatments.^1^, ^3^ Over the past few decades, the development of oral anticancer drugs (OADs), mainly consisting of targeted therapies, has increased substantially.^4^ While targeted OADs offer several advantages over conventional intravenous chemotherapy, such as reduced systemic toxicity and greater ease of administration, they are associated with a higher risk of DDIs due to factors such as chronic use, attainment of steady‐state concentrations, and the involvement of gastrointestinal absorption.^1^, ^5^

Pharmacokinetic (PK) DDIs occur when one drug alters the absorption, distribution, metabolism, or excretion of another drug. Such interactions most commonly involve changes in drug absorption, transport, or modulation of drug‐metabolizing enzymes such as the cytochrome P450 (CYP) enzyme system.^5^ The majority of OADs, including nearly all tyrosine kinase inhibitors (TKIs), are highly metabolized by CYP enzymes, and many are substrates of intestinal, hepatic, renal, and central nervous system drug transporters.^6^, ^7^ Thus, understanding and mitigating the risk of DDIs with OADs is essential to optimizing therapeutic outcomes and minimizing adverse effects in cancer patients.^8^

Potential drug–drug interactions (PDDIs) occur upon the concurrent administration of two or more medications with a high DDI likelihood, regardless of the occurrence of an actual adverse event. Retrospective studies assessing the prevalence of PDDIs among diverse cancer patient populations treated with OADs have produced variable findings, with estimates ranging from as low as 5.4% to as high as 46%.^9^, ^10^, ^11^ This considerable variability can be attributed to study design differences, including differences in follow‐up duration and heterogeneity of the cancer populations included. Discrepancies in PDDI rates among studies also arise from the use of different databases to define interacting drugs (e.g., Micromedex, Lexicomp), since research has demonstrated considerable variability in PDDI rates with OADs among these databases.^12^, ^13^ In addition, PDDIs identified by drug interaction databases are not always clinically relevant, as demonstrated by a recent study that found only ~20% of database‐identified PDDIs were deemed clinically relevant after evaluation by an oncology pharmacist.^14^ Collectively, these findings demonstrate the need for standardization of screening approaches to identify PDDIs and appraise their clinical significance.

The aim of this study was to determine the potential for clinically significant PK‐related DDIs with OADs. Our main objectives included the following: (i) to integrate DDI study findings from US Food and Drug Administration (FDA) drug labels and the primary literature to create a comprehensive list of clinically relevant PK DDI mechanisms for OADs; and (ii) to utilize these mechanisms to determine the prevalence of PK PDDIs involving OADs in a cohort of patients with advanced solid cancers.

## MATERIALS AND METHODS

### Study design and data collection

The Drugs@FDA database^15^ was searched for all new molecular entity approvals with solid or hematologic cancer indications and oral dosage forms prior to January 2023. For each identified OAD, DDI mechanisms were determined by (i) reviewing the original and most recent versions of FDA drug labels and (ii) conducting a primary literature search through PubMed using the following search terms: (“Drug Interactions”[Mesh]) AND *drug generic name* [Supplementary Concept]. FDA labels and PubMed search results were then manually filtered to include only (i) clinical DDI studies designed to assess PK mechanisms, (ii) physiologically‐based pharmacokinetic (PBPK) modeling analyses, (iii) population pharmacokinetic (POPPK) modeling analyses, and (iv) clinical studies that assessed health outcomes. Case reports and *in vitro* studies were not considered sufficiently rigorous for inclusion. Included studies assessed OADs both as object drugs (i.e., their exposure was affected by co‐administered drugs; also known as “victim” drugs) and/or as precipitant drugs (i.e., they affected the exposure of co‐administered drugs; also known as “perpetrator” drugs).

Collected data elements included the following: (i) the OAD and the interacting drug(s); (ii) the mechanism of action of the OAD (summarized in **Table** **S1**); (iii) the FDA recommended clinical management strategy (e.g., co‐administration not recommended); and (iv) drug exposure changes, consisting of observed or predicted geometric mean ratios (GMRs) of the area under the concentration‐time curve (AUC) and/or maximum concentration (*C*_max_) of the object drug during co‐administration with the precipitant drug relative to monotherapy. When AUC data were calculated at multiple time points, the longest interval (e.g., AUC_0‐infinity_) was included in the analysis. When multiple dosing regimens with the same two drugs were used, the strongest exposure change was recorded.

The DDI mechanisms for the interacting drugs (i.e., those other than OADs) were based on the FDA's table of “CYP Enzyme‐ and Transporter‐Based Clinical Substrates, Inhibitors, or Inducers”^16^ and the Indiana University School of Medicine's Flockhart Table.^17^ Assessed DDI mechanisms included (i) inducers, inhibitors, and substrates of the drug‐metabolizing enzymes CYP1A2, CYP2B6, CYP2C8, CYP2C9, CYP2C19, CYP2D6, and CYP3A and (ii) inhibitors or substrates of the major drug transporters listed in **Table** **S2**. DDI mechanisms were assigned to each OAD based on moderate or strong exposure changes, defined as AUC GMRs demonstrating a ≥ 2‐fold but < 5‐fold change (moderate) and ≥ 5‐fold change (strong), during (i) treatment with an inhibitor or substrate of the relevant drug‐metabolizing enzyme or drug transporter, (ii) treatment with strong drug‐metabolizing enzyme inducers (e.g., rifampin), (iii) for acid reducers, treatment with a member of the relevant drug class (i.e., antacids, H2 receptor antagonists [H2RAs], proton pump inhibitors [PPIs]), or (iv) treatment with select individual medications (i.e., imatinib, neomycin). OADs were classified as drug‐metabolizing enzyme or drug transporter substrates based on exposure changes with strong inhibitor drugs (e.g., itraconazole for CYP3A) since inducer drugs commonly utilized in DDI studies (e.g., rifampin) induce multiple phases I and II enzymes. Health outcome studies from the primary literature were also assessed for DDI pairs with putative PK mechanisms, which most commonly consisted of co‐administration of OADs with acid reducers; in these studies, DDI mechanisms were assigned for OADs if changes in health outcomes (e.g., progression‐free survival, overall survival) occurred in patients concurrently receiving both DDI drugs relative to those receiving monotherapy.

FDA clinical recommendations within each drug label were divided into the following categories based on label language, as previously described:^18^ co‐administration contraindicated (CI); co‐administration not recommended (NR); dose adjustment recommended during co‐administration (DA); use with caution during co‐administration (UWC); and no dose adjustment recommended during co‐administration (NDA). When no recommendation was provided, the category of NDA was assumed. If multiple recommendation categories were mentioned, the strongest recommendation was included in our analyses based on the following hierarchy: CI>NR>DA>UWC>NDA. CI, NR, and DA were considered clinically actionable recommendation strategies because each recommends a specific clinical action to mitigate the DDI. To ensure the inclusion of all clinically relevant DDIs in the study, DDI mechanisms were also included if they have an actionable recommendation in the FDA label, regardless of whether the DDI meets the study criteria for exposure change (AUC GMRs ≥ 2‐fold).

### Potential PK DDI prevalence assessment

A retrospective study was conducted in patients treated at the Indiana University Health Precision Genomics Program, a molecular solid tumor clinic and tumor board, and enrolled in the associated research study protocol between 2013 and 2020. The cohort was limited to patients who had ≥ 1 inpatient or outpatient prescription for an OAD; for these patients, all historical medication data were obtained from the Indiana Health Information Exchange, a statewide electronic health record data (EHR) repository. The parent study protocol and an exempt protocol allowing secondary use of Total Cancer Care Protocol data, which supported this study, were approved by the Indiana University Institutional Review Board.

Medication data included date, time, and location (i.e., whether prescribed in an inpatient our outpatient setting or whether dispensed from a pharmacy) for each prescription. In addition, the following data were collected from the EHR for each patient: age, sex, ethnicity, race, and cancer diagnosis. The days' supply for each prescription was estimated using the following assumptions. For prescriptions administered in an inpatient or outpatient setting, the days' supply was assumed to be one. The days' supply for pharmacy‐dispensed prescriptions was based on the shortest duration typically associated with the drug's indicated use (see **Table** **S3** for a complete list of assumed days' supply for pharmacy‐dispensed medications). An exception to this assumption was made for prescriptions dispensed from a pharmacy in regular intervals (e.g., every 30 or 90 days) for medications commonly prescribed as long‐term maintenance therapy for chronic conditions (e.g., antihypertensives); in these cases, it was assumed that the patient continued taking the medication throughout the entire period between consecutive prescriptions.

Within our prevalence analyses, PDDIs were defined as overlapping prescriptions of object and precipitant drugs using the OAD DDI mechanisms classified in our first objective and considering relevant interactions based on CYP and/or transporter annotations from the FDA's table of “CYP Enzyme‐ and Transporter‐Based Clinical Substrates, Inhibitors, or Inducers”^16^ and the Indiana University School of Medicine's Flockhart Table.^17^ For our analyses focused on CYP enzymes and drug transporters, only overlapping prescriptions for moderate or strong inhibitors and inducers with sensitive substrates of the same enzyme or transporter were considered as PDDIs.

### Statistical analyses

Prescription overlap analyses were performed using R Studio (v4.3.1). Continuous data were summarized using mean and standard deviation and compared using two‐sample *t*‐test (if normally distributed) or using medians and interquartile ranges and the Wilcoxon rank‐sum test (if not normally distributed). Normality of distribution was determined using the Kolmogorov–Smirnov goodness‐of‐fit test. Categorical data were summarized by number and percentage and analyzed using the Chi‐square or Fisher's exact test, as appropriate. Statistical comparisons were performed using SAS 9.4 (Cary, NC, USA). For all tests, the prespecified alpha level of significance was *P* < 0.05.

## RESULTS

### PK DDI mechanisms for OADs

In total, 99 OADs were included in the study, of which 53 were TKIs. FDA labels for each OAD and a total of 239 relevant primary literature studies were extracted and reviewed; this resulted in 748 drug–drug pairs (7.5 ± 4.3 [mean ± standard deviation] pairs per OAD) included in the analysis, as summarized in **Appendix** **S1**. PK DDI mechanisms of OADs are summarized in **Table** **1**. Eighty‐five OADs had at least one identified DDI mechanism (85.9% of the included OADs). Victim with metabolic inducers was the most common DDI mechanism, identified in 71 (71.7%) OADs; 33 were victims with both moderate (e.g., efavirenz) and strong (e.g., rifampin) inducers, and 38 were victims with only strong inducers. CYP3A‐related interaction mechanisms were prevalent, with 55 (55.6%) OADs characterized as CYP3A substrates and 29 (29.3%) as CYP3A perpetrators (16 inhibitors and 13 inducers). Additionally, 20 (20.2%) OADs were found to have DDI mechanisms that involved other CYP enzymes, including CYP1A2, CYP2C8, CYP2C9, CYP2C19, and CYP2D6. Drug transporter‐related DDI mechanisms were also identified for 24 (24.2%) OADs, consisting mainly of P‐gp‐ (17) and BCRP‐ (12) related mechanisms. Furthermore, 17 (17.2%) OADs were found to be victims with at least one acid‐reducing agent, including PPIs (17 drugs), H2RAs (9), and antacids (9). The sources of information that informed this study's DDI mechanisms were clinical DDI studies (informed 75.0% of DDI mechanisms), PBPK simulations (41.0%), outcomes studies (2.4%), and POPPK simulations (0.5%); these percentages do not sum to 100% since some DDI mechanisms were informed by multiple sources.

**Table 1:** Identified pharmacokinetic (PK) drug–drug interaction (DDI) mechanisms for oral anticancer drugs

DDI mechanism		Oral anticancer drugs
CYP enzymes		
CYP1A2	Substrates	Erlotinib^a^, Pomalidomide
	Inhibitors	Capmatinib, Enasidenib, Rucaparib, Vemurafenib
CYP2C8	Substrates	Dabrafenib^a^, Enzalutamide, Tucatinib
	Inhibitors	Lapatinib^a^, Pazopanib^a^, Selpercatinib
CYP2C9	Substrates	Erdafitinib^a^
	Inhibitors	Adagrasib, Asciminib, Ceritinib^a^, Rucaparib^a^
	Inducers	Apalutamide^a^, Dabrafenib^a^, Enzalutamide
CYP2C19	Inhibitors	Enasidenib^a^, Rucaparib^a^
	Inducers	Apalutamide, Enzalutamide
CYP2D6	Inhibitors	Abiraterone, Adagrasib, Dacomitinib, Panobinostat^a^, Pazopanib^a^
CYP3A	Substrates	Abemaciclib, Acalabrutinib, Adagrasib, Avapritinib, Axitinib, Bosutinib, Brigatinib, Cabozantinib^a^, Ceritinib, Cobimetinib, Crizotinib, Dabrafenib^a^, Dasatinib, Duvelisib, Elacestrant, Encorafenib, Entrectinib, Erdafitinib^a^, Erlotinib, Everolimus, Futibatinib^a^, Gefitinib, Gilteritinib, Glasdegib, Ibrutinib, Idelalisib^a^, Infigratinib, Ivosidenib, Lapatinib, Larotrectinib, Lorlatinib^a^, Mobocertinib, Neratinib, Nilotinib, Olaparib, Palbociclib^a^, Panobinostat^a^, Pazopanib^a^, Pemigatinib^a^, Pexidartinib^a^, Ponatinib^a^, Pralsetinib, Regorafenib^a^, Relugolix, Ribociclib, Ripretinib, Selpercatinib, Selumetinib^a^, Sonidegib, Sunitinib^a^, Tazemetostat, Toremifene, Vemurafenib^a^, Venetoclax, Zanubritinib
	Inhibitors	Adagrasib, Asciminib^a^, Ceritinib, Crizotinib, Duvelisib, Idelalisib, Imatinib, Lapatinib^a^, Larotrectinib^a^, Nilotinib, Palbociclib^a^, Pazopanib^a^, Ribociclib, Rucaparib^a^, Selpercatinib^a^, Tucatinib
	Inducers	Apalutamide, Belzutifan^a^, Bexarotene, Dabrafenib, Enasidenib^a^, Encorafenib, Enzalutamide, Ivosidenib, Lorlatinib, Mobocertinib^a^, Olutasidenib^a^, Pexidartinib, Sotorasib
Drug transporters		
P‐gp	Substrates	Afatinib^a^, Pazopanib^a^, Pralsetinib, Relugolix^a^, Talazoparib^a^, Venetoclax^a^
	Inhibitors	Adagrasib^a^, Capmatinib^a^, Elacestrant^a^, Gilteritinib^a^, Lapatinib, Selpercatinib^a^, Sotorasib^a^, Tepotinib^a^, Tucatinib^a^, Vemurafenib^a^, Venetoclax^a^
	Inducers	Lorlatinib
BCRP and/or OATP1B1/3	Substrates	Alpelisib^a^, Pazopanib^a^
	Inhibitors	Asciminib^a^, Capmatinib, Darolutamide, Elacestrant^a^, Enasidenib, Encorafenib^a^, Gilteritinib^a^, Regorafenib, Selpercatinib^a^, Sotorasib^a^
MATE1/2K	Inhibitors	Capmatinib^a^
OCT1	Inhibitors	Gilteritinib^a^
UGT	Substrates	Pexidartinib^a^
Victims with inducers		
Strong inducers		Abemaciclib, Abiraterone, Acalabrutinib, Adagrasib, Alectinib, Alpelisib, Avapritinib, Axitinib, Bosutinib, Brigatinib, Cabozantinib, Capmatinib, Ceritinib, Cobimetinib, Crizotinib, Darolutamide, Dasatinib, Duvelisib, Elacestrant, Entrectinib, Encorafenib^a^, Erdafitinib, Erlotinib, Enzalutamide^a^, Everolimus, Exemestane, Futibatinib, Gilteritinib, Gefitinib, Glasdegib, Ibrutinib, Idelalisib, Imatinib, Infigratinib, Ivosidenib^a^, Ixazomib, Larotrectinib, Lapatinib, Lorlatinib, Mobocertinib, Neratinib, Nilotinib, Olaparib, Olutasidenib, Osimertinib, Palbociclib, Panobinostat, Pazopanib^a^, Pemigatinib, Pexidartinib, Pralsetinib, Ponatinib, Regorafenib, Relugolix, Ribociclib, Ripretinib, Selpercatinib, Selumetinib, Sorafenib^a^, Sonidegib, Sotorasib, Sunitinib^a^, Tamoxifen, Tazemetostat^a^, Tivozanib, Toremifene^a^, Tucatinib^a^, Vandetanib^a^, Vemurafenib^a^, Venetoclax, Zanubritinib
Moderate inducers		Abemaciclib, Acalabrutinib, Avapritinib, Brigatinib^a^, Cabozantinib^a^, Capmatinib^a^, Cobimetinib, Darolutamide, Duvelisib^a^, Elacestrant, Entrectinib, Erdafitinib^a^, Erlotinib, Gefitinib, Glasdegib, Ibrutinib, Infigratinib^a^, Larotrectinib, Lorlatinib^a^, Mobocertinib, Neratinib, Olaparib, Olutasidenib^a^, Pemigatinib, Pralsetinib^a^, Ribociclib, Ripretinib, Selpercatinib, Selumetinib^a^, Sonidegib, Tazemetosta^a^, Venetoclax, Zanubritinib
Affected by acid‐reducing agents		
PPI		Acalabrutinib, Bosutinib^a^, Ceritinib, Dasatinib^a^, Dacomitinib^a^, Erlotinib, Gefitinib^a^, Infigratinib^a^, Neratinib, Nilotinib^a^, Palbociclib, Pazopanib^a^, Pexidartinib, Ribociclib, Selpercatinib, Sotorasib, Sunitinib
H2RA		Acalabrutinib, Dasatinib, Erlotinib, Gefitinib^a^, Infigratinib^a^, Nilotinib^a^, Pazopanib^a^, Selpercatinib^a^, Sotorasib^a^
Antacids		Acalabrutinib, Dasatinib, Erlotinib^a^, Gefitinib^a^, Infigratinib^a^, Nilotinib^a^, Pazopanib^a^, Selpercatinib^a^, Sotorasib^a^
Victims with specific drugs		
Victim with tamoxifen		Anastrozole^a^
Victim with gemfibrozil		Bexarotene^a^
Victim with allopurinol		Capecitabine^a^
Victim with neomycin		Sorafenib
Victim with enzalutamide		Talazoparib
No identified DDIs		Bicalutamide, Binimetinib, Decitabine + Cedazuridine, Lenalidomide, Lenvatinib, Letrozole, Niraparib, Selinexor, Thalidomide, Tipiracil + Trifluridine, Trametinib, Umbralisib, Vismodegib, Vorinostat

ARAs, acid‐reducing agents; BCRP, breast cancer resistance protein; CYP, cytochrome P450; H2RA, H2 receptor antagonist; MATE1/2K, multidrug and toxin extrusion protein 1/2K; OATP1B1/3, organic anion transporting polypeptide 1B1/1B3; OCT1, organic cation transporter 1; P‐gp, P‐glycoprotein; PPI, proton pump inhibitor; UGT, uridine 5'‐diphospho‐glucuronosyltransferase.

^a^ Indicates that although ≥ 2‐fold changes in area under the concentration‐time curve were not observed in DDI studies relevant to the specified DDI mechanism, the current FDA label does make an actionable label recommendation.

### Clinically relevant DDI mechanisms without actionable FDA recommendations

The primary literature search identified 16 DDI mechanisms without actionable recommendations in the FDA drug labels for 14 drugs (**Table** **2**), including the following examples. PBPK studies have demonstrated reductions in drug exposure with gefitinib^22^ (AUC GMR of 0.24), ribociclib^23^ (AUC GMR of 0.26), and ibrutinib^24^ (AUC GMR of 0.33) during co‐administration with efavirenz, a moderate CYP3A inducer. While current FDA labels for these drugs make recommendations to avoid concomitant use with strong CYP3A4 inducers, they do not provide recommendations to manage co‐administration with moderate inducers like efavirenz. Similarly, DDI studies have reported a 2.0‐fold decrease in atorvastatin AUC when co‐administered with bexarotene^25^ and a 2.6‐fold increase in midazolam AUC when co‐administered with nilotinib^26^; nevertheless, current FDA labels do not provide recommendations for bexarotene or nilotinib during co‐administration with CYP3A substrates.

**Table 2:** Clinically relevant drug–drug interaction (DDI) mechanisms without actionable FDA recommendations

DDI pair (Precipitant‐object)	Study type	Study results	FDA recommendations
Strong CYP3A inducers‐alectinib	DDI^19^, ^20^	Precipitant: Rifampin AUC GMR: 0.27	None
Moderate CYP3A inducers‐acalabrutinib^a^	PBPK^21^	Precipitant: Efavirenz AUC GMR: 0.39	None (recommendation only mentions strong CYP3A inducers)
Moderate CYP3A inducers‐gefitinibb^a^	PBPK^22^	Precipitant: Efavirenz AUC GMR: 0.24	None (recommendation only mentions strong CYP3A inducers)
Moderate CYP3A inducers‐ribociclib	PBPK^23^	Precipitant: Efavirenz AUC GMR: 0.26	None (recommendation only mentions strong CYP3A inducers)
Moderate CYP3A inducers‐ibrutinib	PBPK^24^	Precipitant: Efavirenz AUC GMR: 0.33	None (recommendation only mentions strong CYP3A inducers)
Bexarotene‐CYP3A substrates	DDI^25^	Object: Atorvastatin AUC GMR: 0.50	None (only mentions hormonal contraceptives)
Nilotinib‐CYP3A substrates	DDI^26^	Object: Midazolam AUC GMR: 2.60	None
Imatinib‐CYP3A substrates	DDI^27^	Object: Simvastatin AUC GMR: 3.50	Use with caution
CYP3A inhibitors‐gefitinibb^a^	PBPK^22^	Precipitant: Darunavir/Ritonavir AUC GMR: 5.50	Use with caution
Moderate CYP3A inhibitors‐ripretinibb^a^	PBPK^28^	Precipitant: Erythromycin AUC GMR: 2.90 Precipitant: Fluconazole AUC GMR: 2.14	Use with caution
PPIs‐palbociclib	Outcomes^29^, ^30^	Reduced PFS during co‐administration with PPIs	None (administration information instructs to take with food)
	DDI^31^	Rabeprazole AUC GMR: 0.38 (fasted conditions); no change under fed conditions	
PPIs‐ribociclibb^a^	Outcomes^30^	Reduced PFS	None
PPIs‐sunitinibb^a^	Outcomes^32^	Reduced PFS and OS, reduced OS	None
PPIs‐ceritinib	DDI^33^	Esomeprazole AUC GMR: 0.24 in healthy subjects and 0.7 in subgroup of patients	None
Regorafenib‐BCRP substrates	DDI^34^	Object: Rosuvastatin AUC GMR: 3.8	Use with caution
Enzalutamide‐ talazoparib	DDI^35^	Object: Talazoparib AUC GMR: 2.00	None

FDA labels were reviewed until 02‐07‐2026 to ensure that no actionable recommendation was included for these drug–drug pairs.

^a^ DDI study was not included in FDA label/review.

### Variability in the DDI mechanisms of OADs within the same therapeutic class

**Figure 1:**
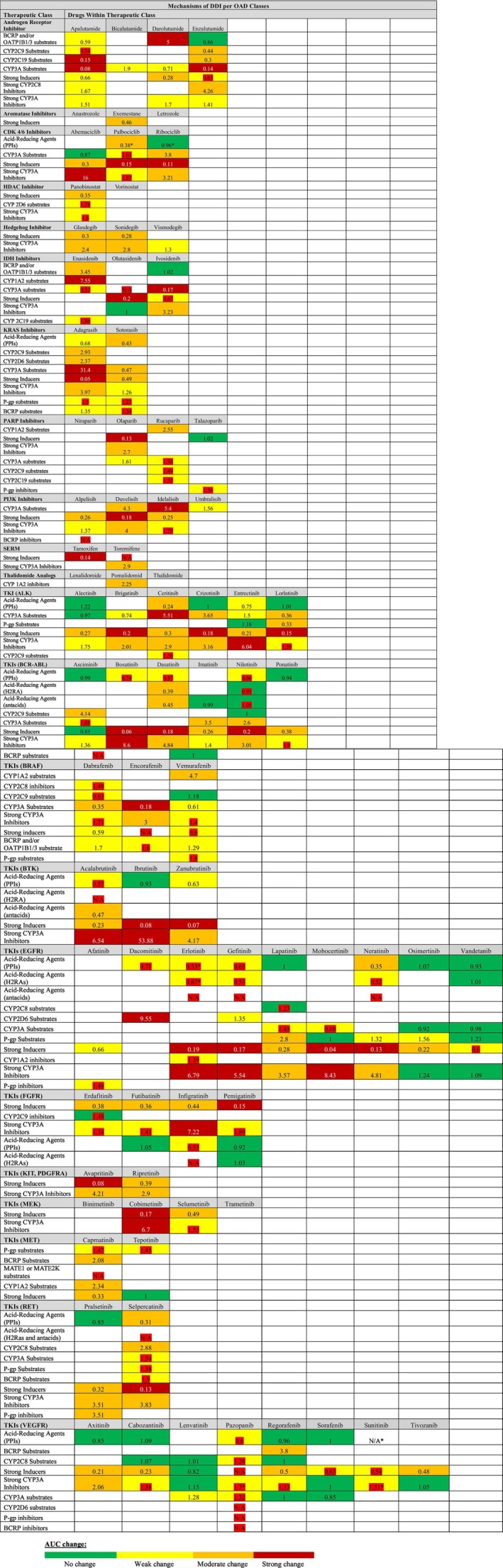
Heat map summarizing the drug–drug interaction (DDI) mechanisms and area under the concentration‐time curve (AUC) changes for each oral anticancer drug (OAD) within therapeutic classes containing at least one OAD. *Indicates that ≥ 1 study demonstrated a change in health outcomes consistent with the putative PK DDI mechanism. (i) For DDIs in which the interacting drug is identified as a “substrate,” the OAD is the precipitant drug; for all other interactions, the OAD is the object. Due to the promiscuity of inducers like rifampin, victims with inducers did not specify a CYP mechanism of effect. (ii) AUC values highlighted in red denote DDI mechanisms that did not meet the prespecified study criterion for exposure change (AUC geometric mean ratio ≥ 2.0) but were considered clinically relevant due to the presence of actionable clinical recommendations in the FDA‐approved labeling.

Our analysis identified variability in the DDI mechanisms of OADs within the same therapeutic class. **Figure** **1** compares the clinically relevant DDI mechanisms (i.e., those with object drug AUC changes ≥ 2‐fold in the presence of the precipitant, significant findings from health outcomes studies, or with actionable FDA recommendations) for each OAD within 22 investigated therapeutic classes with ≥ 1 OAD. For 96 out of the 115 identified clinically relevant DDI mechanisms (83.5%), there was ≥ 1 OAD alternative within the same therapeutic class not involved with the relevant DDI mechanism. The rates of potential substitution for the most common DDI mechanisms were as follows: co‐administration with strong inducers, potential substitution possible for 13 of 21 (62.0%) therapeutic classes; strong CYP3A inhibitors, 13 of 19 (68.4%); CYP3A substrates, 10 of 12 (83.3%); and PPIs, 9 of 9 (100%). Since multiple drugs within the same therapeutic class share similar FDA‐approved indications, differences in DDI mechanisms suggest that therapeutic substitution may be considered to reduce the risk of drug–drug interactions. Accordingly, **Table** **3** outlines the potential for therapeutic substitution by OAD class and indication to avoid clinically relevant DDIs. Although this table provides theoretical suggestions for therapeutic substitution, clinical judgment must be exercised when determining substitution strategies for individual patients.

**Table 3:** Potential therapeutic substation strategies to avoid pharmacokinetic drug–drug interactions (DDI) with oral anticancer drugs (OADs)

OAD class	Indication(s)	Concomitant drugs	Recommended therapeutic substitution
Androgen receptor inhibitors	Prostate cancer	BCRP and/or OATP1B1/3 substrates	Use apalutamide or enzalutamide instead of darolutamide
		CYP2C9 substrates	Use darolutamide instead of apalutamide or enzalutamide
		CYP2C19 substrates	Use darolutamide instead of apalutamide or enzalutamide
		CYP3A substrates	Use darolutamide instead of apalutamide or enzalutamide
		Strong CYP2C8 inhibitors	Use apalutamide or darolutamide instead of enzalutamide
Aromatase inhibitors	Breast cancer	Strong inducers	Use anastrozole instead of exemestane
CDK 4/6 inhibitors	Breast cancer	Acid‐reducing agents (PPIs)	Use abemaciclib instead of palbociclib or ribociclib
		CYP3A substrates	Use abemaciclib instead of palbociclib or ribociclib
Hedgehog inhibitors	Basal cell carcinoma	Strong or moderate inducers	Use vismodegib instead of sonidegib
		Strong or moderate CYP3A inhibitors	Use vismodegib instead of sonidegib
IDH inhibitors	Acute myeloid leukemia	Strong CYP3A inhibitors	Use olutasidenib instead of ivosidenib
KRAS inhibitors	Colorectal cancer, non‐small cell lung cancer	Acid‐reducing agents (PPIs)	Use adagrasib instead of sotorasib
		CYP2C9 substrates	Use sotorasib instead of adagrasib
		CYP2D6 substrates	Use sotorasib instead of adagrasib
		Strong CYP3A inhibitors	Use sotorasib instead of adagrasib
		BCRP substrates	Use adagrasib instead of sotorasib
PARP inhibitors	Breast cancer	Strong or moderate inducers	Use talazoparib instead of olaparib
		Strong or moderate CYP3A inhibitors	Use talazoparib instead of olaparib
	Ovarian cancer	CYP1A2 substrates	Use niraparib or olaparib instead of rucaparib
		Strong or moderate inducers	Use niraparib or rucaparib instead of olaparib
		Strong or moderate CYP3A inhibitors	Use niraparib or rucaparib instead of olaparib
	Prostate cancer	CYP1A2 substrates	Use olaparib or talazoparib instead of rucaparib
		Strong or moderate inducers	Use rucaparib or talazoparib instead of olaparib
		Strong or moderate CYP3A inhibitors	Use rucaparib or talazoparib instead of olaparib
Thalidomide analogs	Multiple myeloma	Strong CYP1A2 inhibitors	Use lenalidomide or thalidomide instead of pomalidomide
TKIs (ALK)	Non‐small cell lung cancer	Acid‐reducing agents (PPIs)	Use alectinib, crizotinib, or lorlatinib instead of ceritinib
		CYP3A substrates	Use alectinib or brigatinib instead of ceritinib, crizotinib, or lorlatinib
		P‐gp substrates	Use ceritinib instead of lorlatinib
		Strong CYP3A inhibitors	Use alectinib instead of brigatinib, ceritinib, crizotinib, or lorlatinib
TKIs (BCR‐ABL)	Chronic myeloid leukemia	Acid‐reducing agents (PPIs, H2RAs, and antacids)	Use imatinib instead of dasatinib or nilotinib
		CYP3A substrates	Use bosutinib or ponatinib instead of imatinib or nilotinib
		Strong CYP3A inhibitors	Use imatinib instead of bosutinib, dasatinib, ponatinib or nilotinib
		Moderate CYP3A inhibitors	Use imatinib or ponatinib instead of bosutinib
TKIs (BRAF)	Melanoma	CYP1A2 substrates	Use dabrafenib or encorafenib instead of vemurafenib
		CYP2C8 inhibitors	Use vemurafenib or encorafenib instead of dabrafenib
		CYP2C9 substrates	Use vemurafenib or encorafenib instead of dabrafenib
		CYP3A substrates	Use vemurafenib instead of dabrafenib or encorafenib
		Strong inducers	Use dabrafenib instead of encorafenib or vemurafenib
		BCRP and/or OATP1B1/3 substrate	Use vemurafenib instead of encorafenib
		P‐gp substrates	Use dabrafenib or encorafenib instead of vemurafenib
TKIs (BTK)	Chronic lymphocytic leukemia/small lymphocytic lymphoma	Acid‐reducing agents (PPIs, H2RAs, and antacids)	Use zanubrutinib (preferred) or ibrutinib instead of acalabrutinib
	Mantle cell lymphoma	Acid‐reducing agents (PPIs, H2RAs, and antacids)	Use zanubrutinib instead of acalabrutinib
TKIs (EGFR)	Breast cancer	Acid‐Reducing Agents (PPIs, H2RAs, and antacids)	Use lapatinib instead of neratinib
		CYP2C8 substrates	Use neratinib instead of lapatinib
		CYP3A substrates	Use neratinib instead of lapatinib
		P‐gp Substrates	Use neratinib instead of lapatinib
	Non‐small cell lung cancer	CYP2D6 substrates	Use afatinib, erlotinib, gefitinib, or osimertinib instead of dacomitinib
		Strong inducers	Use afatinib or dacomitinib instead of erlotinib, gefitinib, or osimertinib
		Strong CYP3A inhibitors	Use osimertinib (preferred), afatinib, or dacomitinib instead of erlotinib or gefitinib
TKIs (MEK)	Melanoma	Strong or moderate inducers	Use binimetinib or trametinib instead of cobimetinib
		Strong or moderate CYP3A inhibitors	Use binimetinib or trametinib instead of cobimetinib
TKIs (MET)	Non‐small cell lung cancer	BCRP substrates	Use tepotinib instead of capmatinib
		MATE1 or MATE2K substrates	Use tepotinib instead of capmatinib
		CYP1A2 substrates	Use tepotinib instead of capmatinib
		Strong inducers	Use tepotinib instead of capmatinib
TKIs (RET)	Non‐small cell lung cancer, thyroid cancer	Acid‐reducing agents (PPIs, H2RAs, and antacids)	Use pralsetinib instead of selpercatinib
		CYP2C8 substrates	Use pralsetinib instead of selpercatinib
		CYP3A substrates	Use pralsetinib instead of selpercatinib
		P‐gp substrates	Use pralsetinib instead of selpercatinib
		BCRP substrates	Use pralsetinib instead of selpercatinib
		P‐gp inhibitors	Use selpercatinib instead of pralsetinib
TKIs (VEGFR)	Gastrointestinal stromal tumors (only if succinate dehydrogenase deficient)	BCRP substrates	Use sunitinib instead of regorafenib
	Hepatocellular carcinoma	BCRP substrates	Use cabozantinib, lenvatinib, or sorafenib instead of regorafenib
		Strong inducers	Use lenvatinib instead of cabozantinib, sorafenib, or regorafenib
	Renal cell carcinoma	BCRP substrates	Use axitinib, cabozantinib, lenvatinib, pazopanib, sorafenib, sunitinib, or tivozanib instead of regorafenib
		Strong inducers	Use lenvatinib instead of axitinib, cabozantinib, regorafenib, tivozanib, sorafenib, pazopanib, or sunitinib
		Strong CYP3A inhibitors	Use, lenvatinib, sorafenib, or tivozanib instead of axitinib, cabozantinib, regorafenib, sunitinib, or pazopanib

The recommendations made in this table are only theoretical based on DDI mechanisms and similar FDA indications and cancer guideline recommendations; clinical judgment, incorporating other patient‐specific factors, should be exercised to determine the appropriateness of OAD therapeutic substitution strategies for individual patients and/or the appropriateness of discontinuing interacting drugs.

BCRP, breast cancer resistance protein; CYP, cytochrome P450; H2RA, H2 receptor antagonist; OATP1B1, solute carrier organic anion transporter family member 1B1; OATP1B3, solute carrier organic anion transporter family member 1B3; P‐gp, P‐glycoprotein 1; PPI, proton pump inhibitor.

### Prevalence of PK PDDIs

A total of 3,697 patients treated at our institutional molecular solid tumor clinic were screened, among whom 1,733 (46.9%) were prescribed ≥ 1 OAD. Of these patients, 454 PDDIs were identified in 302 patients (17.4%). **Table** **4** summarizes the characteristics of patients prescribed OADs with and without PDDIs. The median age at first OAD prescription was similar between groups (PDDI: 60.3 years [Q1–Q3: 51.4–69.4] vs. no PDDI: 59.8 years [50.8–67.4]; *P* = 0.17). Patients with PDDIs were more likely to be male (56.3% vs. 48.3%; *P* = 0.01). Diagnosis at tumor board differed significantly between the two groups; prostate cancer was the most common diagnosis in patients with PDDIs, occurring in 28.1% of these patients, compared with 12.2% of those without PDDIs (standardized residual = 5.9). Similarly, soft tissue sarcoma was overrepresented in patients with PDDIs (15.6% vs. 4.1%, standardized residual = 6.7%). Colorectal cancer, in contrast, was markedly more frequent among patients without PDDIs (15.3% vs. 2.0%; standardized residual = −5.3). PDDI rates for the most common diagnoses at tumor board are provided in the footnote of **Table** **4**. Patients with PDDIs had more concurrent chronic conditions compared with those without PDDIs (64.0% vs. 45.5%, *P* < 0.001) and had numerically higher co‐prescription of all assessed medication classes. In multivariable logistic regression including age, sex, race, ethnicity, and tumor type, only tumor type remained independently associated with PDDIs (*P* < 0.0001), while sex (*P* = 0.67) was no longer significant.

**Table 4:** Demographics characteristics of patients with potential drug–drug interactions (PDDIs) vs. those without PDDIs

Characteristic^a^	Patients with at least one PDDI (*n* = 302)	Patients with no identified PDDIs (*n* = 1,431)	*P*‐value^b^
Age (years)	60.3 (51.4, 69.4)	59.8 (50.8, 67.4)	0.17
Sex			
Male	170 (56.3%)	691 (48.3%)	**0.01**
Ethnicity			
Not Hispanic or Latino	289 (95.7%)	1,385 (96.8%)	0.51
Hispanic or Latino	8 (2.7%)	24 (1.7%)	
Race			
White	269 (89.1%)	1,252 (87.5%)	0.72
Black or African American	24 (8.0%)	123 (8.6%)	
Diagnosis at tumor board^c^			
Prostate	85 (28.1%)	174 (12.2%)	**< 0.001**
Breast	53 (17.6%)	235 (16.4%)	
Soft tissue sarcoma	47 (15.6%)	58 (4.1%)	
Renal	15 (5.0%)	67 (4.7%)	
GIST	13 (4.3%)	15 (1.1%)	
Melanoma	12 (4.0%)	22 (1.5%)	
NSCLC	7 (2.3%)	62 (4.3%)	
Pancreatic	7 (2.3%)	125 (8.7%)	
Colorectal	6 (2.0%)	219 (15.3%)	
Ovarian	3 (1.0%)	47 (3.3%)	
Cholangiocarcinoma	2 (0.7%)	44 (3.1%)	
Other^d^	52 (17.2%)	365 (25.5%)	
Comorbid conditions (*n* = 1,506)^e^			
Any comorbid condition	193 (64.0%)	651 (45.5%)	**< 0.001**
Chronic pain	62 (20.5%)	175 (12.2%)	
Diabetes mellitus	49 (16.2%)	149 (10.4%)	
Heart diseases	37 (12.3%)	103 (7.2%)	
Hypertension	117 (38.7%)	366 (25.6%)	
Hyperlipidemia	72 (23.8%)	236 (16.5%)	
Chronic kidney diseases	25 (8.3%)	91 (6.4%)	
Liver diseases	5 (1.7%)	21 (1.5%)	
Lung diseases	16 (5.3%)	48 (3.6%)	
Neurologic conditions	4 (1.3%)	17 (1.2%)	
Psychiatric conditions	66 (21.9%)	237 (16.6%)	
Co‐prescribed medication classes^f^			
Acid reducers	286 (94.7%)	1,261 (88.1%)	
Antidepressants	207 (68.5%)	829 (57.9%)	
Antidiabetics	140 (46.4%)	562 (39.3%)	
Beta‐blockers	227 (75.2%)	979 (68.4%)	
Diuretics	212 (70.2%)	802 (56.0%)	
NSAIDs	277 (91.7%)	1,162 (81.2%)	
Opioids	299 (99.0%)	1,397 (97.6%)	
RAAS‐acting medications	193 (63.9%)	681 (47.6%)	
Statins	145 (48.0%)	572 (40.0%)	
Thyroid supplements	84 (27.8%)	319 (22.3%)	

GIST, gastrointestinal stromal tumors, NSAIDs, non‐steroidal anti‐inflammatory drugs; NSCLC, non‐small cell lung cancer; RAAS, renin‐angiotensin‐aldosterone system; Statins, β‐hydroxy‐β‐methylglutaryl coenzyme A reductase inhibitors.

^a^ For continuous variables, the median (Q1, Q3) is displayed. For categorical variables, the count (%) is displayed.

^b^ *P*‐values < 0.05 are bolded.

^c^ PDDI rates by diagnosis at tumor board were as follows: GIST (46.4%), soft tissue sarcoma (44.8%), melanoma (35.3%), prostate cancer (32.8%), breast (18.4%), renal (18.3%), NSCLC (10.1%), ovarian (6.0%), pancreatic (5.3%), cholangiocarcinoma (4.3%), and colorectal cancers (2.7%).

^d^ The “other” diagnosis at tumor board category includes all diagnoses with ≤ 25 patients in the cohort.

^e^ Diagnosis data were available for 1,506 patients in our cohort (87%), consisting of 277 in our PDDI cohort and 1,229 in our non‐PDDI cohort. Comorbid conditions were assessed only for patients with available diagnosis date and were defined as chronic diseases, categorized as follows: heart diseases (heart failure, ischemic heart disease, arrhythmias, and conduction disorders), liver diseases (cirrhosis, fibrosis, chronic hepatitis), chronic lung disease (asthma, COPD, emphysema), neurologic conditions (Parkinson's disease, Alzheimer's disease, epilepsy), and psychiatric conditions (anxiety, depression, bipolar disorder, schizophrenia).

^f^ Co‐prescription was defined as ≥ 1 prescription for a drug in the relevant medication class.

**Figure 2:**
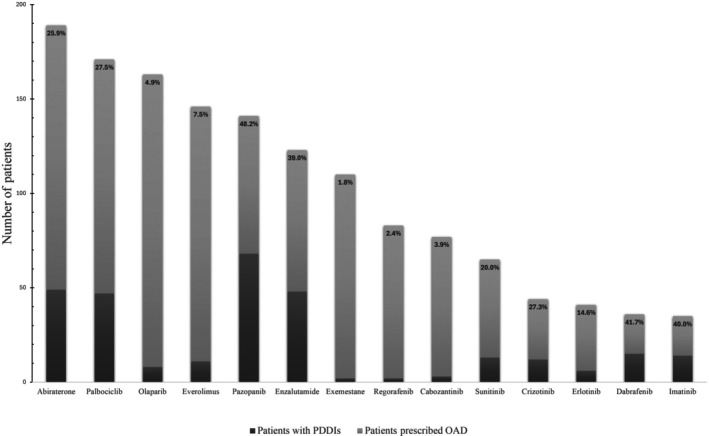
Patient‐level potential drug–drug interaction (PDDI) prevalence for the most commonly prescribed oral anticancer drugs (OADs).

The number of PDDIs involving OADs ranged from 1 to 7 per patient, with 1, 2, and 3 PDDIs identified in 11.7%, 3.9%, and 1.0% of patients, respectively. The most common OAD DDI mechanisms in patients with ≥ 1 PDDI were as follows: CYP3A perpetrators (58.9%); CYP2D6 perpetrators (32.5%); CYP3A substrates (18.9%); victims with PPIs (14.6%); CYP2C19 perpetrators (6.6%); and CYP2C9 perpetrators and victims with H2RAs (both 7.7%). The patient‐level PDDI prevalence and DDI mechanisms for the most commonly prescribed OADs are illustrated in **Figure** **2**. Notably, 48.2% of patients prescribed pazopanib experienced at least 1 PDDI; the other OADs that were prescribed to ≥ 35 patients with the highest PDDI prevalence were dabrafenib (41.7%), imatinib (40.0%), and enzalutamide (39.0%). The patient‐level PDDI prevalence of all OADs, based on the DDI mechanism, is provided in **Appendix** **S2**. The most common interacting drugs, along with their mechanisms of interaction with OADs, are shown in **Figure** **S1**.

## DISCUSSION

In this study, we identified the clinically relevant PK DDI mechanisms for 99 FDA‐approved OADs. To the best of our knowledge, this is the first analysis summarizing the PK DDI mechanisms of all OAD classes based on FDA drug labels and the primary literature. We then utilized these DDI mechanisms to assess the prevalence of PK PDDIs in a cohort of patients with advanced solid tumors receiving OADs. Our findings demonstrate that the majority of OADs had ≥ 1 DDI mechanism, with CYP3A‐related interaction mechanisms being the most frequent. In addition, 17.4% of our cohort experienced ≥ 1 PK PDDI involving an OAD.

Our findings demonstrate that FDA reviews are generally effective at identifying DDI mechanisms and communicating clinical strategies to manage these interactions. Of the 153 DDI mechanisms that resulted in ≥ 2‐fold change in AUC, the corresponding FDA label recommendations were clinically actionable in 137 (89.5%) of these cases. In the 16 cases without actionable recommendations, four had actionable recommendation with strong inducers only, despite the ≥ 2‐fold decrease in AUC with moderate inducers.^21^, ^22^, ^23^, ^24^ Similarly, bexarotene reduced the AUC of atorvastatin, a CYP3A substrate, by 2‐fold; however, FDA recommendations included DDI management recommendation only with hormonal contraceptives.^25^ In addition, four DDI mechanisms, consisting of imatinib with CY3A substrates,^27^ CYP3A inhibitors with gefitinib,^22^ moderate CYP3A inhibitors with ripretinib,^28^ and regorafenib with BCRP substrates,^34^ had FDA recommendations of increased monitoring or to use with caution only, although a ≥ 2‐fold increase in AUC was observed.^22^, ^27^, ^28^, ^34^ Two DDI mechanisms, ribociclib^30^ and sunitinib^32^ with PPIs, were extracted from outcomes studies and were not included in the FDA labels. The last five DDI mechanisms were included in the FDA labels but had no actionable FDA recommendations, four of which had seemingly appropriate reasons for not requiring DDI management, which included the following: (i) the AUC was within the bioequivalence range when considering parent alectinib plus active metabolites during co‐administration with rifampin^19^, ^20^; (ii) the object (talazoparib) and precipitant (enzalutamide) are indicated in combination, with efficacy and safety demonstrated in clinical trials^35^; (iii) one clinical trial subgroup of non‐small cell lung cancer patients (DDI determined by POPPK modeling) showed only minimal changes in ceritinib AUC during co‐administration with esomeprazole^33^; and (iv) the DDI mechanism only resulted in palbociclib AUC reductions in the fasted state (as confirmed by clinical study data) during co‐administration with PPIs, and the FDA label instructs that palbociclib be taken with food.^31^ For palbociclib, it is noteworthy that two retrospective studies have found reduced progression‐free survival during co‐administration with PPIs, suggesting that real‐world patients may not be following the FDA recommendation to take with food.^29^, ^30^ While the rationale is less apparent for the remaining cases, factors such as sufficiently broad therapeutic indices may account for the lack of actionable DDI recommendations within these FDA labels.

A clinically relevant finding from our study is that for most OAD therapeutic classes, the drugs within each class have variable DDI mechanisms. Given that there are frequently multiple drugs within each class with similar FDA‐approved indications, this variability in DDI mechanisms raises the possibility of therapeutic substitution to mitigate DDI risk. For example, the CDK 4/6 inhibitors ribociclib and palbociclib are both victims with strong inducers, CYP3A substrates, moderate CYP3A inhibitors,^23^, ^31^ and subject to DDIs with acid reducers.^(^ ^29^, ^30^, ^31^ ^)^ Abemaciclib, on the other hand, is similarly a victim with strong inducers and a CYP3A substrate,^36^ but is not a CYP3A inhibitor^37^ and is not expected to exhibit pH‐sensitive absorption subject to reductions by acid‐reducing agent therapy.^36^ Given the interchangeable efficacy of these agents,^38^, ^39^ breast cancer patients requiring concomitant therapy with acid reducers or sensitive CYP3A substrates could thus be treated with abemaciclib.

Our findings indicate that the prevalence of PK PDDIs in patients with advanced solid cancers prescribed OADs is 17.4%. Kim *et al*.^10^ reported a prevalence of 26.4% of PDDI in a retrospective cross‐sectional study that included 11,076 patients with cancer prescribed OADs; PDDIs were identified using 2 DDI databases, Lexicomp and Micromedex. Similarly, in a cohort of 898 ambulatory cancer patients diagnosed with solid tumors or hematologic malignancies and receiving OADs, van Leeuwen *et al*.^11^ reported a PDDI prevalence of 46%, identified using the DDI database Facts and Comparisons. We believe our reported PDDI prevalence is lower than that of previous studies for two main reasons: (i) while prior studies analyzed both PK and PD mechanisms, we focused solely on PK mechanisms, which directly impact drug exposure and thereby therapeutic efficacy and the risk of toxicity; and (ii) we adopted a conservative methodology for DDI classification, including only (i) moderate or strong DDIs (defined by AUC GMR changes of ≥ 2‐fold) based on clinical DDI studies, PBPK and POPPK modeling analyses, (ii) significant findings from health outcomes studies, or (iii) actionable FDA label recommendations; this methodology excluded less rigorous evidentiary sources like case reports and *in vitro* studies. While our methodology diminishes the importance of the maximum concentration (*C*_max_), another important parameter of drug disposition that is known to be correlated with the efficacy and toxicity of certain drugs, we believe that in general, using AUC alone better captured clinically meaningful changes in drug exposure while determining that the two parameters were highly correlated in our dataset (i.e., for the 156 DDI pairs where the *C*_max_ GMR was ≤ 0.5 or ≥ 2, the AUC GMR was also ≤ 0.5 or ≥ 2 in 141 (90.4%) of these cases). Altogether, we believe this conservative methodology, along with careful manual curation of outcomes studies in the literature, more specifically captures clinically relevant PK DDIs compared with the DDI software‐based assessments used in previous studies. While our study population consisted exclusively of patients with advanced solid cancers, differing from the broader cohorts investigated in prior studies, we expect that this difference would actually increase the PDDI rate in our population relative to comparators, since advanced cancer patients are known to have an especially high rate of polypharmacy.^40^, ^41^ Finally, the patients in our study received care by a multidisciplinary medical team that included oncologists and pharmacists with expertise in DDI management, who may have clinically intervened to lower the prevalence of PDDIs in our study cohort.

Our analyses suggest that patients with certain cancer types are at increased risk for PDDIs. These findings are supported by recent literature highlighting the complexity of pharmacological management in patients with sarcoma and prostate cancer.^42^, ^43^, ^44^ Given that these cancers predominantly affect older adults, sarcoma and prostate cancer patients are frequently at increased DDI risk due to medical comorbidities necessitating complex medication regimens.^42^, ^43^ Additionally, the use of TKIs targeting numerous kinases, androgen receptor inhibitors, and CYP17A1 inhibitors increases the risk of DDIs in these populations since all of these classes exhibit multiple CYP‐related DDI mechanisms. For instance, pazopanib is a substrate of CYP3A, an inhibitor of CYP2C8, CYP2D6, and CYP3A, and a victim with strong inducers;^45^ enzalutamide is a strong CYP3A inducer, a moderate inducer of CYP2C9 and CYP2C19, a CYP2C8 substrate, and a victim with strong inducers;^35^ and abiraterone is a moderate CYP2D6 inhibitor and a victim with strong inducers.^46^

We acknowledge several limitations of our analyses assessing PDDI prevalence with OADs. First, our cohort consists solely of advanced solid cancer patients, which (i) precluded the assessment of PDDI prevalence for drugs with only hematologic indications and (ii) limited assessment of PDDI prevalence for drugs with both solid cancer and hematologic indications (e.g., imatinib) to solid cancer indications. Second, the analysis of PDDI prevalence was based on prescription orders collected from a statewide EHR data repository, which does not capture medication adherence and excludes the potential for PDDIs involving over‐the‐counter medications. Third, the dataset used in our analysis only contained inpatient and outpatient medication data until October 2020; thus, OADs that were approved after that time were not included in our analysis. Fourth, we included all OADs approved through January 2023, excluding newer agents. Finally, our analysis was limited to assessing PDDIs (i.e., temporal overlap of prescriptions for object and precipitant drugs) since our data did not allow for assessment of the clinical relevance of identified PDDIs using health outcomes data. Future studies evaluating clinical outcomes would provide a more comprehensive understanding of the health impact of these DDIs.

In conclusion, our study thoroughly analyzed the primary literature and FDA drug labels for 99 OADs, identifying a comprehensive list of clinically relevant PK DDI mechanisms and an estimate of the prevalence of PK PDDIs in a cohort of patients with advanced solid cancers. Our findings demonstrate that PK DDI mechanisms are abundant and that PK PDDIs are common with OADs, underscoring the need for careful monitoring and management of DDIs in this at‐risk patient population.

## FUNDING

This research was supported by National Institute of General Medical Sciences awards R35GM145383 (ZD) and K23GM147805 (TS).

## CONFLICTS OF INTEREST

All authors declared no competing interests for this work.

## AUTHOR CONTRIBUTIONS

F.A., I.R.Y., S.I.J., T.F.L., R.M., S.K.Q., Z.D., T.C.S., J.E.T., S.M.B., and T.S. wrote the manuscript and designed the research; F.A. and T.S. performed the research and analyzed the data.

## Supporting information


Data S1

Appendix S1

Appendix S2
