# Supplementary material for: Pharmacokinetic Drug–Drug Interaction Potential of Oral Anticancer Drugs
Source: Clin Pharmacol Ther. 2026 Mar 7;119(6):1614–27. doi: 10.1002/cpt.70253 (PMC13156348; doi:10.1002/cpt.70253)
Supplement: Supplementary file 1 — Data S1 [file CPT-119-1614-s002.docx]

**Supplementary Tables**

**Table S1. Categorization of mechanisms of action for OADs included in the study.**

| **Generic Name** | **Mechanism of Action** |
| --- | --- |
| Abemaciclib | CDK 4/6 Inhibitor |
| Abiraterone | CYP 17 Inhibitor |
| Acalabrutinib | TKI (BTK) |
| Adagrasib | KRAS Inhibitor |
| Afatinib | TKI (EGFR) |
| Alectinib | TKI (ALK) |
| Alpelisib | PI3K Inhibitor |
| Anastrozole | Aromatase Inhibitor |
| Apalutamide | Androgen Receptor Inhibitor |
| Asciminib | TKI (BCR-ABL) |
| Avapritinib | TKI (PDGFRA, KIT) |
| Axitinib | TKI (VEGFR) |
| Belzutifan | HIF2A Inhibitor |
| Bexarotene | RXR Activator |
| Bicalutamide | Androgen Receptor Inhibitor |
| Binimetinib | TKI (MEK) |
| Bosutinib | TKI (BCR-ABL) |
| Brigatinib | TKI (ALK) |
| Cabozantinib | TKI (VEGFR) |
| Capecitabine | Antimetabolite |
| Capmatinib | TKI (MET) |
| Ceritinib | TKI (ALK) |
| Cobimetinib | TKI (MEK) |
| Crizotinib | TKI (ALK) |
| Dabrafenib | TKI (BRAF) |
| Dacomitinib | TKI (EGFR) |
| Darolutamide | Androgen Receptor Inhibitor |
| Dasatinib | TKI (BCR-ABL) |
| Decitabine; Cedazuridine | Nucleoside Metabolic Inhibitor; Cytidine Deaminase Inhibitor |
| Duvelisib | PI3K Inhibitor |
| Elacestrant | Selective Estrogen Receptor Degrader |
| Enasidenib | IDH2 Inhibitor |
| Encorafenib | TKI (BRAF) |
| Entrectinib | TKI (ALK) |
| Enzalutamide | Androgen Receptor Inhibitor |
| Erdafitinib | TKI (FGFR) |
| Erlotinib | TKI (EGFR) |
| Everolimus | mTOR Inhibitor |
| Exemestane | Aromatase Inhibitor |
| Futibatinib | TKI (FGFR) |
| Gefitinib | TKI (EGFR) |
| Gilteritinib | TKI (FLT3) |
| Glasdegib | Hedgehog Inhibitor |
| Ibrutinib | TKI (BTK) |
| Idelalisib | PI3K Inhibitor |
| Imatinib | TKI (BCR-ABL) |
| Infigratinib | TKI (FGFR) |
| Ivosidenib | IDH1 Inhibitor |
| Ixazomib | Proteasome Inhibitor |
| Lapatinib | TKI (EGFR) |
| Larotrectinib | TKI (TRK) |
| Lenalidomide | Thalidomide Analog |
| Lenvatinib | TKI (VEGFR) |
| Letrozole | Aromatase Inhibitor |
| Lorlatinib | TKI (ALK) |
| Mobocertinib | TKI (EGFR) |
| Neratinib | TKI (EGFR) |
| Nilotinib | TKI (BCR-ABL) |
| Niraparib | PARP Inhibitor |
| Olaparib | PARP Inhibitor |
| Olutasidenib | IDH1 Inhibitor |
| Osimertinib | TKI (EGFR) |
| Palbociclib | CDK 4/6 Inhibitor |
| Panobinostat | HDAC Inhibitor |
| Pazopanib | TKI (VEGFR) |
| Pemigatinib | TKI (FGFR) |
| Pexidartinib | TKI (CSF1R, KIT, FLT3) |
| Pomalidomide | Thalidomide Analog |
| Ponatinib | TKI (BCR-ABL) |
| Pralsetinib | TKI (RET) |
| Regorafenib | TKI (VEGFR) |
| Relugolix | GnRH Receptor Antagonist |
| Ribociclib | CDK 4/6 Inhibitor |
| Ripretinib | TKI (PDGFRA, KIT) |
| Rucaparib | PARP Inhibitor |
| Selinexor | Nuclear Export Inhibitor |
| Selpercatinib | TKI (RET) |
| Selumetinib | TKI (MEK) |
| Sonidegib | Hedgehog Inhibitor |
| Sorafenib | TKI (VEGFR) |
| Sotorasib | KRAS Inhibitor |
| Sunitinib | TKI (VEGFR) |
| Talazoparib | PARP Inhibitor |
| Tamoxifen | Selective Estrogen Receptor Modulator |
| Tazemetostat | Methyltransferase Inhibitor |
| Tepotinib | TKI (MET) |
| Thalidomide | Thalidomide |
| Tipiracil; Trifluridine | Nucleoside Metabolic Inhibitor; Thymidine Phosphorylase Inhibitor |
| Tivozanib | TKI (VEGFR) |
| Toremifene | Selective Estrogen Receptor Modulator |
| Trametinib | TKI (MEK) |
| Tucatinib | TKI (HER2) |
| Umbralisib | PI3K Inhibitor |
| Vandetanib | TKI (EGFR) |
| Vemurafenib | TKI (BRAF) |
| Venetoclax | BCL-2 Inhibitor |
| Vismodegib | Hedgehog Inhibitor |
| Vorinostat | HDAC Inhibitor |
| Zanubritinib | TKI (BTK) |

**Table S2. Drug transporters included in the analysis.**

| **Transporter** | **Abbreviation** | **Gene** |
| --- | --- | --- |
| Breast cancer resistance protein | BCRP | ***ABCG2*** |
| Multidrug and toxin extrusion protein 1 | MATE1 | ***SLC47A1*** |
| Multidrug and toxin extrusion protein 2 | MATE2-K | ***SLC47A2*** |
| Organic anion transporter 1 | OAT1 | ***SLC22A6*** |
| Organic anion transporter 3 | OAT3 | ***SLC22A8*** |
| Solute carrier organic anion transporter family member 1B1 | OATP1B1 | ***SLCO1B1*** |
| Solute carrier organic anion transporter family member 1B3 | OATP1B3 | ***SLCO1B3*** |
| Organic cation transporter 1 | OCT1 | ***SLC22A1*** |
| Organic cation transporter 2 | OCT2 | ***SLC22A2*** |
| P-glycoprotein 1 | P-gp | ***ABCB1*** |

**Table S3. Assumed days supply by medication for prescriptions dispensed from a pharmacy (used to determine concomitant administration of OADs with interacting drugs to determine potential drug-drug interactions).**

| **Generic Name** | **Days Supply** |
| --- | --- |
| Abemaciclib | 30 |
| Abiraterone | 30 |
| Afatinib | 30 |
| Alectinib | 30 |
| Allopurinol | 30 |
| Alosetron | 30 |
| Alpelisib | 30 |
| Alprazolam | 7 |
| Aluminum Hydroxide; Magnesium Hydroxide | 14 |
| Amiodarone | 7 |
| Amlodipine | 30 |
| Amphetamine | 30 |
| Anastrozole | 30 |
| Apalutamide | 30 |
| Atorvastatin | 30 |
| Axitinib | 30 |
| Bicalutamide | 30 |
| Binimetinib | 30 |
| Brigatinib | 30 |
| Bupropion | 30 |
| Buspirone | 30 |
| Cabozantinib | 30 |
| Caffeine | 7 |
| Calcium Carbonate | 14 |
| Capecitabine | 14 |
| Carbamazepine | 30 |
| Carisoprodol | 7 |
| Carvedilol | 30 |
| Cimetidine | 14 |
| Citalopram | 30 |
| Clarithromycin | 7 |
| Clopidogrel | 30 |
| Codeine | 7 |
| Crizotinib | 30 |
| Dabigatran | 30 |
| Dabrafenib | 30 |
| Darolutamide | 30 |
| Desipramine | 30 |
| Dexamethasone | 7 |
| Digoxin | 30 |
| Diphenhydramine | 1 |
| Dronabinol | 1 |
| Duloxetine | 30 |
| Edoxaban | 30 |
| Encorafenib | 30 |
| Enzalutamide | 30 |
| Erdafitinib | 30 |
| Erlotinib | 30 |
| Erythromycin | 7 |
| Escitalopram | 30 |
| Everolimus | 30 |
| Exemestane | 30 |
| Famotidine | 14 |
| Famotidine; Ibuprofen | 14 |
| Fenofibrate | 30 |
| Fentanyl | 7 |
| Fexofenadine | 30 |
| Fluconazole | 7 |
| Fluoxetine | 30 |
| Flurbiprofen | 7 |
| Fluvoxamine | 30 |
| Gemfibrozil | 30 |
| Glyburide | 30 |
| Ibuprofen | 7 |
| Imatinib | 30 |
| Isoniazid | 30 |
| Ixazomib | 15 |
| Ketoconazole | 7 |
| Lansoprazole | 14 |
| Lapatinib | 30 |
| Larotrectinib | 30 |
| Lenalidomide | 21 |
| Lenvatinib | 30 |
| Letrozole | 30 |
| Magnesium Oxide | 14 |
| Meclizine | 1 |
| Melatonin | 30 |
| Meloxicam | 7 |
| Methadone | 7 |
| Methylprednisolone | 7 |
| Metronidazole | 7 |
| Mirtazapine | 30 |
| Neomycin | 7 |
| Neratinib | 30 |
| Nifedipine | 30 |
| Nilotinib | 30 |
| Niraparib | 30 |
| Nizatidine | 14 |
| Olanzapine | 30 |
| Olaparib | 30 |
| Omeprazole | 14 |
| Ondansetron | 2 |
| Osimertinib | 30 |
| Oxcarbazepine | 30 |
| Oxycodone | 7 |
| Palbociclib | 30 |
| Pantoprazole | 14 |
| Paroxetine | 30 |
| Pazopanib | 30 |
| Phenobarbital | 30 |
| Pioglitazone | 30 |
| Piroxicam | 7 |
| Pitavastatin | 30 |
| Pomalidomide | 21 |
| Pravastatin | 20 |
| Prednisone | 7 |
| Promethazine | 1 |
| Raloxifene | 30 |
| Ramelteon | 30 |
| Ranitidine | 14 |
| Regorafenib | 30 |
| Repaglinide | 30 |
| Ribociclib | 30 |
| Risperidone | 30 |
| Rosuvastatin | 30 |
| Rucaparib | 30 |
| Sertraline | 30 |
| Simvastatin | 30 |
| Sirolimus | 30 |
| Sodium Bicarbonate | 14 |
| Sorafenib | 30 |
| Sulfasalazine | 30 |
| Sunitinib | 30 |
| Tacrolimus | 30 |
| Tamoxifen | 30 |
| Terbinafine | 30 |
| Theophylline | 30 |
| Tipiracil; Trifluridine | 12 |
| Tizanidine | 30 |
| Topiramate | 30 |
| Toremifene | 30 |
| Torsemide | 30 |
| Trametinib | 30 |
| Trazodone | 30 |
| Trimethoprim; Sulfamethoxazole | 7 |
| Vandetanib | 30 |
| Vemurafenib | 30 |
| Venlafaxine | 30 |
| Vismodegib | 30 |
| Warfarin | 30 |

**Supplementary Figures**

**Figure S1. Most common interacting drugs with oral anticancer drugs (OADs), along with the mechanisms of interaction.**

**Note**: The denominator is the number of PDDIs of each mechanism
